# The Use of Internal States to Guide Behavior Is Associated with Functional Engagement of the Anterior Insula in Male Rats

**DOI:** 10.1523/ENEURO.0156-25.2025

**Published:** 2025-08-08

**Authors:** Mickaël Puaud, Dhaval D. Joshi, Alfie Wright, Victor Ho, Aude Belin-Rauscent, Maxime Fouyssac, Barry J. Everitt, Yvan Peterschmitt, David Belin

**Affiliations:** ^1^Department of Psychology, University of Cambridge, Cambridge CB2 3EB, United Kingdom; ^2^Department of Physiology, Development and Neuroscience, University of Cambridge, Cambridge CB2 3EB, United Kingdom; ^3^Université Marie et Louis Pasteur, UMR INSERM 1322 LINC, Besançon F-25000, France

**Keywords:** anterior insular cortex, drug discrimination task, interoception, isoproterenol, pentylenetetrazol, Zif268

## Abstract

Interoception and associated subjective states shape adaptive behaviors. In humans, interoceptive information is hierarchically processed in the insular cortex (IC), being integrated first in the posterior IC (PIC) and then processed in the anterior IC (AIC) to generate subjective states. However, it has not been established whether this is the case in other species nor whether utilization of interoceptive states to guide behavior is also specifically associated with functional engagement of the AIC, as suggested by this hierarchical model. We investigated in male Sprague Dawley rats whether the use of pharmacologically induced internal states to guide instrumental behavior in a discrimination task functionally engages the AIC as opposed to the mere experience of such states. Rats trained to use the interoceptive state produced by the centrally acting GABA_A_ receptor antagonist pentylenetetrazol (PTZ) or the peripherally acting β-adrenoreceptor agonist isoproterenol to guide their behavior performed as well in a discrimination task as those trained to use an exteroceptive visual discriminative stimulus. While interoceptive internal states were as potent as exteroceptive cues to guide instrumental behavior, only the former were associated with an increase in mRNA levels of the cellular plasticity marker, zif268 in the PIC, as assessed using qPCR. In contrast, zif268 mRNA levels increased in the AIC only after rats had used PTZ-induced interoceptive states to guide behavior, not simply in response to PTZ administration. These results show that in rats, the utilization of interoceptive states to guide behavior is associated with functional engagement of the AIC.

## Graphical abstract

**Figure:**
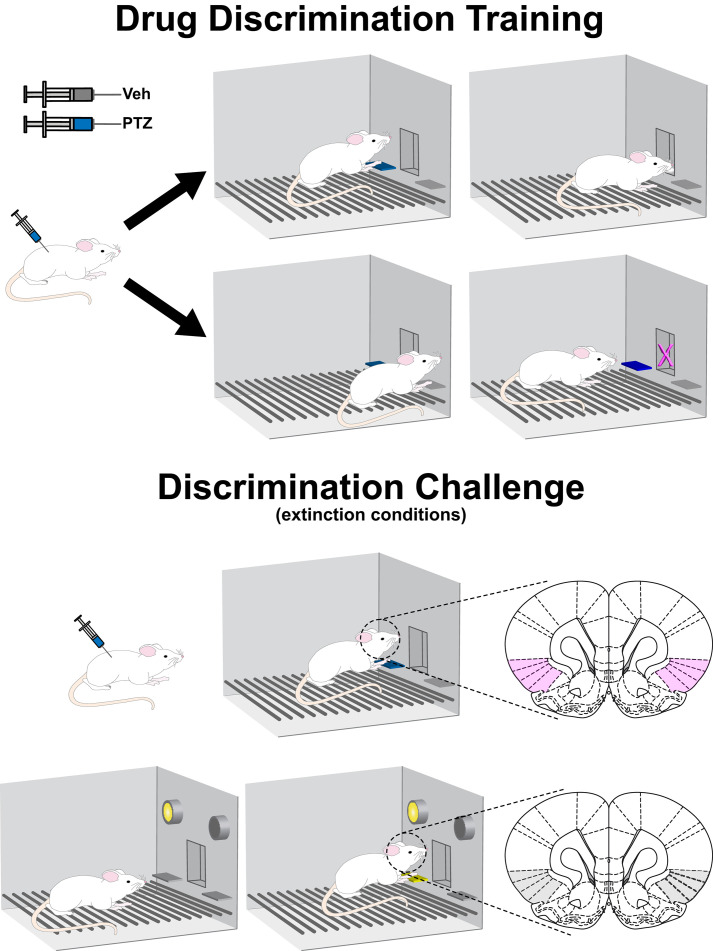
Visual Abstract

## Significance Statement

In humans, interoceptive information is hierarchically processed in the insula, being integrated first in the posterior insula and then processed in the anterior insula to generate subjective states. However, it has not been established whether this is the case in other species nor whether the utilization of interoceptive states to guide behavior is also specifically associated with functional engagement of the anterior insula. In the present study, we demonstrate that interoceptive states can be used as discriminative stimuli by rats as effectively as exteroceptive cues to guide their instrumental behavior via different underlying neural systems. Central detection of internal states and their utilization to guide instrumental behavior engage cellular plasticity in the PIC and the AIC, respectively.

## Introduction

Interoception, the process of sensing, appraising, and producing mental representations of internal physiological states (Sherrington, 1906; Craig, 2002, 2003), influences behavior through its contribution to emotions, motivation, and executive functions such as emotion regulation, decision-making, and impulse control (Liu et al., 2007; Lee et al., 2008; Weller et al., 2009; Li et al., 2010; Jones et al., 2011; Lopez-Larson et al., 2012; Churchwell and Yurgelun-Todd, 2013; Garfinkel and Critchley, 2013; Critchley and Garfinkel, 2017; Rae et al., 2019; Zamariola et al., 2019; Lischke et al., 2020).

At the neural systems level, interoception critically depends on the insular cortex (IC). Patients with lesions encompassing, or resections of, the IC are unable to report internal states (Ronchi et al., 2015) or urges (Naqvi et al., 2007; Naqvi and Bechara, 2010), and they are impaired in decision-making tasks (Augustine, 1996; Clark et al., 2008; Von Siebenthal et al., 2017; Wang et al., 2019). Within the IC, functional subdomains are involved in distinct facets of interoception and interoceptive control over behavior (Tataranni et al., 1999; Egan et al., 2003; Critchley et al., 2004; Zaki et al., 2012; Hassanpour et al., 2016; Wang et al., 2019). In humans, the posterior IC (PIC) is considered to integrate a primary map of the interoceptive signals transduced from the sensory properties of a particular stimulus (Craig, 2009; Hassanpour et al., 2016). The anterior IC (AIC), on the other hand, is functionally engaged during interoceptive awareness and when interoceptive signals are used in higher emotional and cognitive functions, such as emotion regulation or decision-making (Craig, 2002). For example, the AIC is engaged when bottom-up interoceptive signals serve as cues to guide ongoing behavior (Craig, 2009; Kurth et al., 2010) or when top-down mechanisms call for a representation of future states to which the AIC contributes by generating priors—or somatic markers (Damasio, 1996)—that shape behavior.

In rodents, bottom-up interoceptive states have been shown causally to generate affective states, such as anxiety, via activation of the PIC (Hsueh et al., 2023), replicating previous evidence in humans (Hassanpour et al., 2018). Similarly, the AIC has also been shown in rodents to influence the ability to maximize reward in cost–benefit decision-making tasks (Daniel et al., 2017) and impulse control (Lopez-Larson et al., 2012; Belin-Rauscent et al., 2016). This suggests that a similar PIC→AIC integration of interoceptive control over behavior exists across species.

However, whether a similar PIC/AIC dichotomy exists in the utilization of internal states to guide motivated behavior remains to be elucidated. Here we used a drug discrimination task (Huang and Ho, 1974; Gianutsos and Lal, 1975; Colpaert et al., 1976; Vellucci et al., 1988; Young, 2009) to investigate the extent to which the experience of pharmacologically induced peripherally or centrally generated internal states versus their utilization to guide instrumental behavior are associated with differential functional engagement of the AIC and the PIC.

To this end, we capitalized on the study by Vellucci et al. (1988), which showed using the drug discrimination task that the discriminative stimulus properties of the internal state produced by the centrally acting GABA_A_ antagonist pentylenetetrazol (PTZ) generalized to the state produced by stressors such as social defeat. These results provided clear evidence that pharmacologically evoked states can substitute for ethologically relevant internal states, such as anxiety, and be utilized by animals to guide behavior. However, since interoception is bidirectional, having both body to brain bottom-up (Hsueh et al., 2023) and brain to body top-down mechanisms (Berntson and Khalsa, 2021; Chen et al., 2021), it is not known whether the peripherally generated internal states that otherwise produce affective states (Hsueh et al., 2023) can be used as effectively as exteroceptive and centrally mediated interoceptive discriminative stimuli to guide motivated, instrumental behaviors. Rats were trained to use the discriminative properties of exteroceptive cues, i.e., cue lights, or a peripherally acting β-adrenoceptor agonist with positive chronotropic effects isoproterenol (ISO) or the centrally acting PTZ. The reliance on the learned internal states to guide instrumental responding was then tested under extinction conditions. The neural systems functionally recruited selectively by the experience of interoceptive states, or their utilization to guide behavior, as opposed to that of exteroceptive discriminative stimuli (Madangopal et al., 2019), were identified by analysis of the level of expression of the plasticity marker zif268 (Magnard et al., 2024) using qPCR.

## Materials and Methods

### Animals

Male Sprague Dawley rats (*n* = 42, Charles River) weighing ∼300 g upon arrival were housed in pairs for a week of habituation to the temperature-controlled vivarium (20–23°C) under a 12 h reverse light/dark cycle (lights off at 07:00 A.M.) with food and water available *ad libitum*. Rats were then single-housed and food-restricted to 85–90% of their theoretical free-feeding body weight. Experiments were performed during the dark phase, 6–7 d per week, and were conducted in accordance with the United Kingdom 1986 Animals (Scientific Procedures) Act following ethical review by the University of Cambridge Animal Welfare and Ethical Review Body (AWERB) under the project license number 70/8072 held by David Belin.

### Procedures

**Figure 1.**
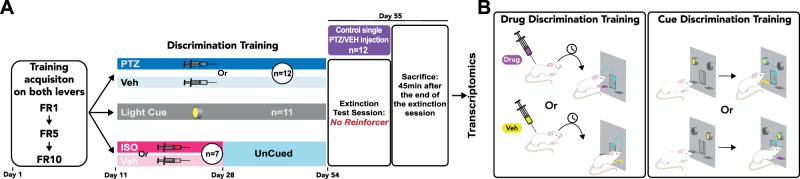
Timeline of the experiments and drug discrimination task (DDT) design. ***A***, Timeline of the experiment. Rats were to press either of two available levers for 45 mg food pellets under a fixed ratio 1 schedule of reinforcement (one press on either lever resulted in the delivery of a pellet, FR1). The active lever alternated between the left and the right lever each day. The fixed ratio schedule of reinforcement was then progressively increased to FR5 and then FR10, under which rats were trained until they obtained at least 25 reinforcers with each lever. The rats were trained in discrimination tasks under three different conditions: one group to discriminate PTZ versus VEH, one group to discriminate light cues, and one group to first discriminate ISO versus VEH for 18 d (until day 28) and used as an UnCued control group until the end of the discrimination training. After the training was complete (54 d, 44 d of discrimination), rats were challenged for the ability to discriminate the different cues under extinction conditions. Forty-five minutes after the end of the session, rats were killed and their brains were harvested and fresh frozen. Brains were then processed into 300-μm-thick coronal sections, and tissue samples were µ-punched from brain regions of interest for mRNA level quantification by qPCR. Another cohort of rats received an injection of PTZ or VEH without prior training to investigate the selective functional engagement of the AIC and the PIC by the utilization of an interoceptive state to guide behavior versus its passive experience. Rats were first trained to respond on both levers independently under serially increasing fixed ratio schedules: FR1, FR5, and then FR10. ***B***, Discrimination tasks. The rats then progressed onto drug or cue discrimination training over 44 daily sessions. In these sessions, both levers were presented, but on any single day, responding on only one of these levers—the active lever—was now reinforced. For each rat, the active lever for any given training session was pseudorandomly selected to be the left or the right lever from one session to the next. Thus, for a rat to respond on the correct lever on any given session, it had to use either the discriminative properties of an interoceptive stimulus (PTZ, *n* = 12, or ISO, *n* = 12, vs VEH) administered intraperitoneally 20 min before the start of the session or that of an exteroceptive white light cue (LightCue) illuminated above the active lever throughout the session. For drug discrimination, a specific interoceptive discriminative stimulus was always paired with the same active lever, randomly allocated across individuals (Fig. 1*B*). For instance, VEH was always paired with the right lever and PTZ with the left for a given rat; it was the opposite for another rat. During the initial stages of learning (the first 10 daily sessions) for each group, the different discrimination conditions (PTZ vs VEH, ISO vs VEH or LightCue vs no cue). Then, to increase unpredictability, the drug rats received on any given day (thereby determining which of the two levers was the active one) followed a pseudorandom sequence so that rats never received PTZ or ISO on 2 consecutive days, but never had more than four sessions between two PTZ or ISO administrations. (FR, fixed ratio; ISO, isoproterenol; PTZ, pentylenetetrazol; VEH, vehicle).

As illustrated in the schematic timeline of the experiments in Figure 1*A*, after habituation to the animal facility, rats were trained in Med Associates chambers instrumentally to press either of two available levers for 45 mg food pellets (Bio-Serv) under fixed ratio schedules of reinforcement for 10 d. Rats were then trained either under a drug discrimination or visual discrimination task for 44 d, at the end of which their discrimination performance was assessed over a 12 min challenge session under extinction conditions (no more pellet delivery upon lever press; Vellucci et al., 1988). Forty-five minutes after this challenge session, rats were killed, and their brains were harvested fresh, snap frozen, and stored at −80°C until processed for qPCR alongside those of an untrained control group. This group was referred to as “Passive,” which comprised 12 drug-naive rats killed 45 min after an intraperitoneal injection of either PTZ (*n* = 6) or vehicle (*n* = 6). The PTZ-administered rats of this control group had passively experienced the interoceptive effects of PTZ, unlike the PTZ-trained group (referred to as “Active”), which utilized them as a discriminative stimulus to guide their instrumental reward-related behavior.

### Drugs

Pentylenetetrazol (PTZ, Sigma) and isoproterenol (ISO, LKT Laboratories), dissolved in sterile 0.9% NaCl (Henry Schein) vehicle (VEH), were administered intraperitoneally (IP; 0.5 ml/kg) at a concentration of 20 and 5 mg/kg, respectively (Vellucci et al., 1988; Kemp and Manahan-Vaughan, 2008). Control treatment consisted of injections of equal volume of VEH.

### Apparatus

Experiments were conducted in 12 standard operant chambers (Med Associates) controlled by Med-PC software (Med Associates). Chiefly, each chamber (29.5 × 32.5 × 23.5 cm), housed in a ventilated, sound-attenuating cubicle, consisted of aluminum sidewalls and clear polycarbonate ceiling, front and back walls anchored to a grid floor. Two retractable levers (4 cm wide) were situated 8 cm above the grid floor and 12 cm apart; a white cue light (2.5 W, 24 V) was situated above each lever and a white house light (2.5 W, 24 V) on the wall opposite the levers. A food magazine, connected to a food dispenser controlled by Med-PC, was situated between the two levers, 2 cm above the grid floor allowing the automatic delivery of 45 mg food pellets (Bio-Serv).

### Drug discrimination task

Training in the drug discrimination task was carried out similarly to the procedure described in Vellucci et al. (1988). In order to avoid hyponeophagia, rats were first habituated in their home cage to thirty 45 mg food pellets. The following day, rats were habituated to the operant chambers for 30 min, during which they had access to 30 pellets previously placed in the magazine. Then, rats were habituated to pellet deliveries in the food magazine over a 15 min session during which pellets were delivered under a variable interval 30 (VI30) schedule. Rats were subsequently trained over six sessions (two per day for 3 d) lasting 15 min each to press either of two available levers for 45 mg food pellets (Bio-Serv) under a fixed ratio 1 schedule of reinforcement (one press on either lever resulted in the delivery of a pellet). The active lever alternated between the left and the right lever each day, and rats eventually obtained 30 reinforcers in a single session for each lever.

The fixed ratio schedule of reinforcement was then progressively increased to FR5 (2 sessions, 1 d) and then FR10 (6 sessions, 6 d), under which rats were trained until they obtained at least 25 reinforcers with each lever. The contingency was maintained at FR10 for the rest of the experiment under discrimination training.

The rats then progressed onto drug discrimination training, which consisted of 44 daily sessions of 12 min each, carried out 6–7 d per week over ∼7 weeks. In these sessions, both levers were presented, but on any single day, responding on only one of these levers—the active lever—was now reinforced. For each rat, the active lever for any given training session was pseudorandomly selected to be the left or the right lever from one session to the next.

Thus, for a rat to respond on the correct lever on any given session, it had to use the discriminative properties of an interoceptive stimulus (PTZ, *n* = 12, or ISO, *n* = 12, vs VEH) administered intraperitoneally 20 min before the start of the session or that of an exteroceptive white light cue (LightCue) illuminated above the active lever throughout the session. For drug discrimination, a specific interoceptive discriminative stimulus was always paired with the same active lever, randomly allocated across individuals (Fig. 1*B*). For instance, VEH was always paired with the right lever and PTZ with the left for a given rat; it was the opposite for another rat. During the initial stages of learning (the first 10 daily sessions) for each group, the different discrimination conditions (PTZ vs VEH, ISO vs VEH or LightCue vs no cue) were administered in alternating order (i.e., day 1 PTZ, day 2 VEH, day 3 PTZ etc.) to habituate the animal to daily changes in the discriminative stimulus. Then, to increase unpredictability, the drug rats received on any given day (thereby determining which of the two levers was the active one) followed a pseudorandom sequence so that rats never received PTZ or ISO on 2 consecutive days, but never had more than four sessions between two PTZ and ISO administrations.

#### Discrimination training

During the drug discrimination training sessions, responding on the inactive lever (IL) prior to the completion of each FR10 sequence resulted in the reset of the AL counter, thereby delaying procurement of the reinforcer. Each next trial started only after the food pellet delivered upon completion of a sequence of 10 uninterrupted AL presses was collected by the animal. Because rats would necessarily know which lever is active on any session upon receipt of the first reinforcer, irrespective of the discriminative stimuli they were trained to rely on, each daily drug discrimination session began with a 30 s (±15 s) variable interval (VI) during which lever presses were not reinforced. This enabled the daily measurement of the bias toward responding on the lever signaled as active by the discriminative stimulus, which was computed as the discrimination index: (AL_until first reward_) / (AL_until first reward_ + IL_until first reward_) × 100. When no active or inactive lever press was performed, the learning criterion was computed as 0.

Because of a suspected adverse side effect of ISO in one individual, treatment had to be discontinued for this group after 18 sessions and six other rats were discarded to ensure that the behavioral outcomes presented were not confounded by any noninteroceptive effect of isoproterenol. This did not prevent us from demonstrating the ability rats show to use peripherally generated interoceptive states to guide behavior as revealed by the comparison of the learning discrimination criterion under ISO to that shown once ISO was no longer administered, i.e., in the absence of any discriminative cue (“UnCued” condition).

#### Discrimination challenge

After 44 training sessions, the cued groups (PTZ-trained and LightCue) had achieved a stable level of discrimination performance under training conditions, namely, the first uninterrupted FR10 sequence on the correct lever was achieved after the VI30 period in at least 8 out of 10 sessions. The reliance on the discriminative cues to guide responding was subsequently assessed in a 12 min challenge session under extinction conditions. PTZ-trained animals received an intraperitoneal injection of either PTZ or VEH (*n* = 6 each). All of the “UnCued” animals received an intraperitoneal injection of VEH (*n* = 7). Finally, the “LightCue”-trained rats (*n* = 11) were challenged with either the right cue/lever or the left cue/lever at test. Six of these animals that were subsequently used in qPCR experiments received a vehicle injection like all the other control groups. In addition, an additional cohort of 12 rats received an injection of either PTZ or VEH (*n* = 6 each) without prior drug discrimination training in order subsequently to compare the neural signature of a simple experience of the PTZ- versus VEH-induced state to that of the use of these states to guide motivated behavior. For the animals exposed to the 12 min challenge, the accuracy factor was calculated: 100 × (AL + 1) / (AL + IL + 1) over the first 6 min of the session, since responding either decreases or generalizes to both levers afterward in the absence of reinforcement (Vellucci et al., 1988). However, given that accuracy is not related to response rates under these conditions (Vellucci et al., 1988), the observation that some individuals persisted in responding on the correct lever for longer periods of time led us to assess the persistence of the reliance on discriminative cues to guide responding over time. This was done by comparing the accuracy factor achieved in the first and last 6 min of the session with the persistence index calculated: (Accuracy during the last 6 min / Accuracy during the first 6 min) × 100.

### Tissue collection

Forty-five minutes following the challenge session, rats were deeply anesthetized with isoflurane and decapitated. Brains were harvested, snap frozen in −40°C (±5°C) isopentane (Sigma-Aldrich), and stored at −80°C until their processing for qPCR.

### Micropunching and RNA extraction

Bilateral micropunches of the AIC (AP = 3–0; ML = 3–7; DV = −5 to −8), the PIC (AP = 0 to −3; ML = 5.2–7.2; DV = −6.3 to −8), the basolateral (BLA; AP = −2 to −3.3; ML = 4.7–5.7; DV = −8 to −9.1), and central (CeA; AP = −1.6 to −2.8; ML = 3.2 to −4.9 ; DV = −7.6 to −8.7) territories of the amygdala, which had previously shown to be involved in the discriminative properties of PTZ (Vellucci et al., 1988) and M1 motor cortex (M1; AP = 3.6–1.5; ML = 2–5.1; DV = −0.8 to −3.8), intended to be used as a negative control, were performed on 300-µm-thick coronal brain sections processed with a cryostat (Leica) at −15°C with a sterile 1 mm biopsy trocar based on the stereotactic atlas of the rat brain (Paxinos and Watson, 2006). Micropunches were then placed in sterile 1.5 ml Eppendorf tubes and stored at −80°C until further processing. Samples were then homogenized, and RNA was extracted as previously described (Marti-Prats et al., 2021) using a “Quick-RNA MicroPrep” (Zymo) according to manufacturer's instructions. Samples were treated with DNase I during this process, and finally the RNA levels were quantified using a NanoDrop spectrophotometer (Thermo Fisher Scientific).

### Quantitative PCR

After RNAs were reverse-transcribed into cDNAs with the “RT2 first strand Kit” (Qiagen) according to the manufacturer's instructions, real-time PCR was performed as previously described (Chernoff et al., 2025). Chiefly, qPCR was carried out on the CFX96 Real-Time PCR Detection System (Bio-Rad) using the RT2 SYBR Green Mastermix (Qiagen) under the following conditions: reaction volume of 25 μl (1 μl cDNA, 24 μl PCR reaction mixture), 1 initial step at 95°C (10 min) followed by 40 temperature cycles (95°C for 15 s, 60°C for 60 s). Primers were used to assess the level of zif268 (*egr1*, PPR44272B) mRNA relative to the housekeeping gene cyclophilin A (peptidylprolyl isomerase A, *ppia*, PPR06504A). Cyclophilin A was selected as the housekeeping gene for ubiquitous tissue normalization since it was confirmed not to be affected by PTZ across brain regions in a previous pilot experiment. Gene-specific amplification was verified using a melting curve analysis. The relative mRNA level of the target gene was calculated using CFX Manager Software (Bio-Rad) and expressed as 2-ΔCT (Livak and Schmittgen, 2001) relative to cyclophilin A.

### Data and statistical analyses

Data, analyzed using STATISTICA 10 (StatSoft), are presented as mean ± SEM or box plots [median ± 25% (percentiles) and Min/Max as whiskers]. Assumptions for normal distribution, homogeneity of variance, and sphericity were verified using the Shapiro–Wilk, Cochran C, and Mauchly's sphericity test, respectively.

Accuracy at challenge was analyzed using one-way and repeated-measures analysis of variance (ANOVA) using lever presses emitted during the first half of the session or over the first and second halves (used as within-subject factors), respectively. Experimental groups, namely, PTZ-trained, ISO/UnCued, and LightCue were used as between-subject factor.

Discrimination learning under reinforcement (e.g., discrimination index) was analyzed using a repeated-measures ANOVA with sessions as within-subject factor and groups (PTZ-trained, ISO/UnCued, and LightCue) as between-subject factor. Because of between-session variability in performance across groups, data pertaining to the acquisition of discrimination were analyzed across 12, 3-d blocks. Since the discontinuation of ISO treatment occurred on the last session of the 6th 3 d block, the effect of the loss of this peripherally generated interoceptive discriminative stimulus on behavior was assessed by comparing performance across the six blocks before and after the “ISO” group transitioned to the “UnCued” condition.

The relative expression of zif268 was analyzed for the following four structures: AIC, PIC, M1, BLA, and CeA using a factorial ANOVA with mRNA level ratios as within-subject factor and groups and drug at test (rats trained to use PTZ to guide their behavior (PTZ-trained) challenged under PTZ (PTZ-active) or Vehicle (VEH-active) and rats experiencing the effects of PTZ versus Vehicle passively, namely, passive-PTZ versus passive-VEH) as between-subject factors. These analyses were performed on Log-transformed data as assumptions of normality were slightly violated for the analysis of M1 (Passive-PTZ and PTZ-trained), the AIC, and the CeA, while the assumption of homogeneity of variance was violated for the PIC and BLA data.

Significant interactions were analyzed further using Bonferroni’s post hoc analyses or planned comparisons wherever appropriate.

Pearson's *r* was used to assess the relationship between the learning criterion and the accuracy at challenge as well as the relationship between the levels of mRNA in the AIC and the persistence index.

For all statistical analyses, significance was set at *α* = 0.05. Effect sizes are reported as partial eta squared (*pη*^2^).

## Results

### Rats learnt equally to discriminate exteroceptive and centrally or peripherally generated interoceptive cues

**Figure 2.**
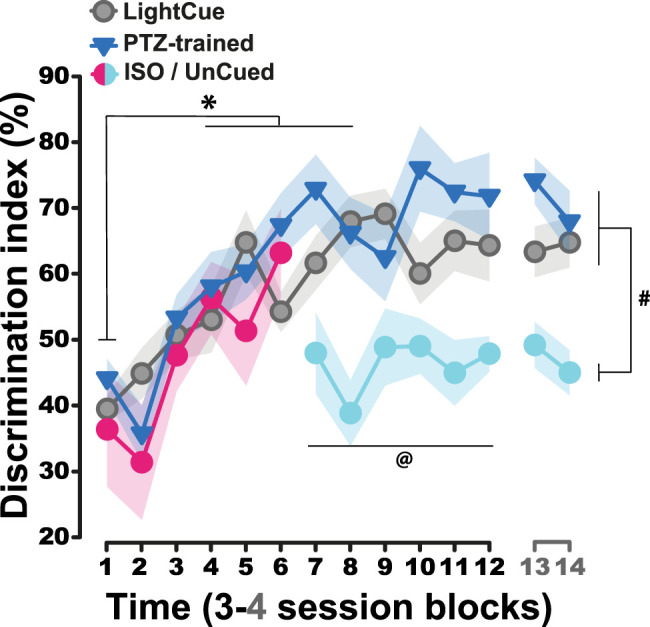
Rats learnt equally to discriminate exteroceptive and centrally or peripherally generated interoceptive cues. The discrimination index, which represents response allocation guided by the discriminative stimulus prior to the delivery of the first reinforcer, increased equally for all the groups over the first period of 24 d (6 blocks or 4 d; main effect of blocks: *F*_(5,135)_ = 11.12, *p* < 0.0001, *pη*^2^ = 0.29, group: *F*_(2,27)_ < 1, *p* = 0.51, *pη*^2^ = 0.15 and group × block interaction: *F*_(10,135)_ = 1.33, *p* = 0.22, *pη*^2^ = 0.09) so that by block 4 their performance differed from that on block 1 (*p*s < 0.04). However, when rats that were trained to use the discriminative properties of isoproterenol no longer received any discriminative stimulus (and hence were switched to the “UnCued” condition) on block 7, their performance fell to ∼50% (i.e., chance level; planned comparison block 6 vs subsequent blocks: *F*_(1,6)_ = 6.55, *p* < 0.05) while that of both groups trained under the control of a discriminative stimulus, namely, PTZ-trained and LightCue groups, remained at ∼70% (main effect of group: *F*_(2,27)_ = 7.22, *p* = 0.003, *pη*^2^ = 0.35, first 6 vs last 6 blocks: *F*_(1,27)_ = 10.73, *p* = 0.003, *pη*^2^ = 0.29; and group × first 6 vs last 6 blocks interaction: *F*_(2,27)_ = 8.34, *p* = 0.002, *pη*^2^ = 0.38, ^#^ planned comparison of the PTZ-trained and LightCue groups vs the ISO/UnCued group across the first 6 blocks vs last 6 blocks: *F*_(1,27)_ = 16.412, *p* < 0.001). Thus, while during the first 6-block period, none of the groups differed from each other, during the second 6-block period, the two groups trained under the control of a discriminative stimulus maintained the same level of performance as they had achieved at the end of the first period and differed from ISO/Uncued group (post hoc test: UnCued vs PTZ-trained and Uncued vs LightCue, *p*s < 0.02). ISO, isoproterenol; PTZ, pentylenetetrazol. *: versus block 1, *p* < 0.05. ^#^: Uncued versus the two groups trained under the control of a discriminative stimulus, *p* < 0.05. ^@^: Uncued group, versus block 6, *p* < 0.05.

All rats learnt equally to utilize exteroceptive and interoceptive cues to guide their instrumental responses over the first six blocks of drug discrimination training sessions (Fig. 2), as revealed by a similar increase in the learning criterion score in all groups (main effect of blocks: *F*_(5,135)_ = 11.12, *p* < 0.0001, *pη*^2^ = 0.29, group: *F*_(2,27)_ < 1, *p* = 0.51, *pη*^2^ = 0.15 and group × block interaction: *F*_(10,135)_ = 1.33, *p* = 0.22, *pη*^2^ = 0.09). By block 6, discrimination performance had reached a plateau in all groups, which was maintained until the end of training in the PTZ-trained and LightCue groups. However, performance decreased to chance levels in the ISO group as soon as the rats were no longer presented with a discriminative stimulus (UnCued condition) (main effect of group: *F*_(2,27)_ = 7.22, *p* = 0.003, *pη*^2^ = 0.35, first 6 vs last 6 blocks: *F*_(1,27)_ = 10.73, *p* = 0.003, *pη*^2^ = 0.29; and group × first 6 vs last 6 blocks interaction: *F*_(2,27)_ = 8.34, *p* = 0.002, *pη*^2^= 0.38), thereby resulting in a clear difference in discrimination performance between the PTZ-trained and LightCue groups on the one hand and the ISO/UnCued group on the other hand (planned comparison of the two groups trained under discriminative stimulus control, namely PTZ and LightCue vs the ISO/UnCued group across the first 6 blocks vs last 6 blocks: *F*_(1,27)_ = 16.412, *p* < 0.001).

### The salience of interoceptive discriminative stimuli is associated with the ability to adaptively guide instrumental behavior over long periods of time

**Figure 3.**
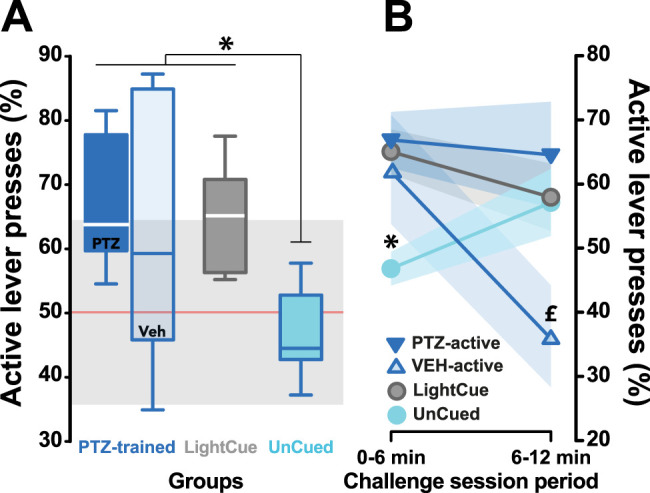
The salience of interoceptive discriminative stimuli is associated with the ability to adaptively guide instrumental behavior over longer periods of time. ***A***, During a 12 min challenge session under extinction conditions, discrimination accuracy, expressed as the percentage of active lever presses [(active lever presses / total level presses) × 100], was much higher in the two groups trained under the control of a discriminative stimulus, namely, the PTZ-trained and LightCue groups, than the Uncued control group that performed at chance throughout the session (main effect of group: *F*_(3,21)_ = 3.44, *p* = 0.035, p*η*^2^ = 0.33, planned comparison of the two the two groups trained under the control of a discriminative stimulus vs UnCued group: *F*_(1,21)_ = 9.87, *p* < 0.01). ***A***, ***B***, During the first 6 min of the challenge session, the rats trained to use the interoceptive discriminative properties of PTZ to guide their behavior challenged under vehicle (VEH-active, light blue) or PTZ (PTZ-active, deep blue) as well as the LightCue (gray) groups displayed much higher accuracy than the UnCued control group. ***B***, However, while the LightCue and PTZ-active rats maintained a high discrimination performance throughout the entire session (first vs last 6 min of the 12 min challenge session) VEH-active rats progressively switched to the PTZ-paired lever, thereby performing at a much lower level of accuracy than the other two groups during the second half of the session (UnCued vs PTZ-active during the first 6 min, * post hoc: *p* < 0.05). The light gray shading represents the performance theoretically governed by chance (50% accuracy ± standard deviation of animals across all groups). PTZ, pentylenetetrazol; VEH, vehicle. (* Uncued vs the two groups trained under the control of a discriminative stimulus, namely, PTZ-trained and LightCue groups, post hoc: *p* < 0.05; ^£^ 6 first minutes vs 6 last minutes, post hoc: *p* < 0.05).

After 44 d of discrimination training, the ability of each rat to utilize a specific discriminative stimulus to guide its motivated instrumental behavior was assessed under extinction conditions. Over the first 6 min of the challenge session, PTZ-trained rats displayed a similar level of performance as those using an exteroceptive discriminative stimulus, i.e., LightCue-trained rats in that both groups showed a much higher discrimination performance than that of the UnCued group, which performed at chance level (Fig. 3*A*; main effect of group: *F*_(3,21)_ = 3.44, *p* = 0.035, *pη*^2^ = 0.33, planned comparison of the two groups trained under discriminative stimulus control vs UnCued group: *F*_(1,21)_ = 9.87, *p* < 0.01). Remarkably, unlike LightCue- and PTZ-trained rats having received PTZ at test, PTZ-trained rats challenged with VEH at test tended to switch their response to the inactive PTZ-associated lever during the final 6 min period of the 12 min challenge session, thereby demonstrating a less sustained reliance on the discriminative properties of the relatively less salient VEH-induced interoceptive state (Fig. 3*B*; main group × period interaction: *F*_(3,21)_ = 5.74, *p* < 0.01, *pη*^2^ = 0.45, planned comparison of both, PTZ-active and LightCue, vs VEH-active × period interaction: *F*_(1,21)_ = 7.18, *p* ≤ 0.01). Post hoc analyses revealed that while the performance displayed by PTZ-active and LighCue groups did not change across periods, that of the VEH-active profoundly decreased (*p* < 0.05).

### The utilization of internal states to guide behavior is specifically associated with functional recruitment of the AIC

**Figure 4.**
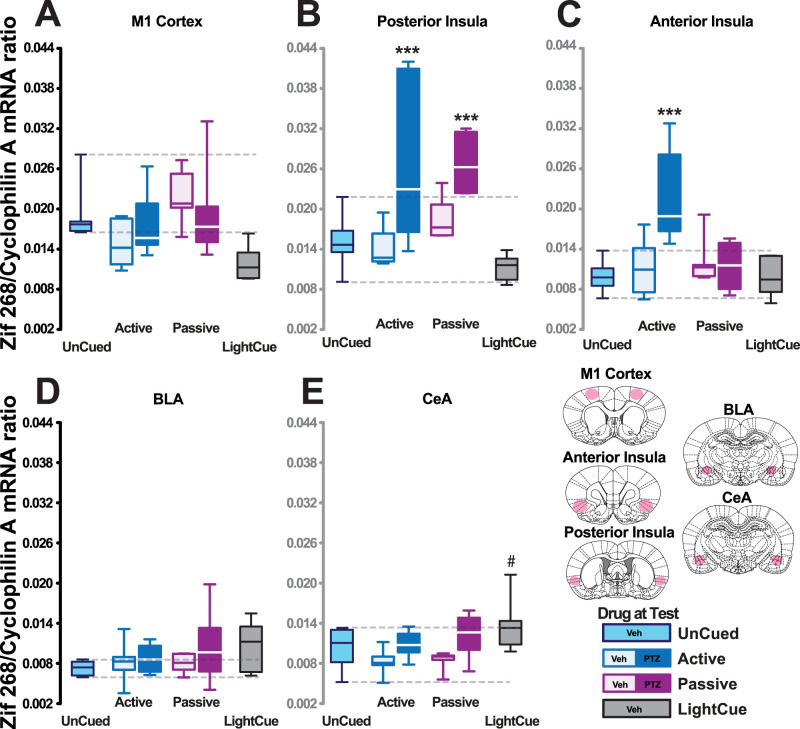
The utilization of internal states to guide behavior is specifically associated with a functional recruitment of the AIC. ***A***, PTZ administration did not evoke an increase of zif268 mRNA in the M1 cortex (planned comparison between the PTZ-treated groups vs the rest: *F*_(1,30)_ = 1.44, *p* = 0.24). ***B***, An increase in zif268 mRNA levels was observed in the PIC after exposure to PTZ in both the passive and active conditions (main effect of group: *F*_(5,31)_ = 10.09, *p* < 0.001, *pη*^2^ = 0.61, planned comparison between PTZ-treated vs all other conditions: *F*_(1,31)_ = 39.40, *p* < 0.001). ***C***, In marked contrast, in the AIC, Zif268 mRNA levels were increased only when rats used the discriminative stimulus properties of PTZ to guide their instrumental behavior, namely, in the active condition (main effect of group: *F*_(5,31)_ = 5.68, *p* < 0.001, *pη*^2^ = 0.48, *** vs all the other groups, post hoc test, *p*s < 0.04). Such an effect was not observed in the PTZ-treated animals in the BLA (***D***; main effect of group: *F*_(5,31)_ = 0.85, *p* = 0.52, *pη*^2^ = 0.12) or the CeA (***E***) in which the LightCue group differed from the VEH-active and VEH-passive groups (main effect of group: *F*_(5,31)_ = 3.19, *p* = 0.019, *pη*^2^ = 0.36, ^#^ vs VEH-passive and VEH-active groups post hoc tests, *p*s < 0.03).

The differential neural signature of the reliance on exteroceptive or PTZ- or VEH-evoked interoceptive discriminative stimuli to guide instrumental behavior (“Active”) versus the untrained control group (“Passive”) was then investigated by assessing the mRNA levels of the plasticity marker zif268 (Knapska and Kaczmarek, 2004) in the network involving the anterior and posterior territories of the interoceptive cortex, namely, the AIC and the PIC as well as the two functional domains of the amygdala, which is necessary for PTZ discrimination (Vellucci et al., 1988), namely, the BLA and the CeA. PTZ administration did not result in an overall, nonspecific increase in zif268 mRNA levels (Fig. 4*A*), as shown by the absence of difference between the two PTZ-treated groups and the other experimental groups in zif268 mRNA levels in the M1 cortex (planned comparison: *F*_(1,30)_ = 1.44, *p* = 0.24).

PTZ resulted in an increase in zif268 mRNA levels in the PIC in rats that used its discriminative stimulus properties to guide their behavior (PTZ-trained-Active-PTZ) and untrained controls (Passive-PTZ), which only experienced its effect passively (main effect of group: *F*_(5,31)_ = 10.09, *p* < 0.001, *pη*^2^ = 0.61, planned comparison between PTZ-treated vs all other conditions: *F*_(1,31)_ = 39.40, *p* < 0.001; Fig. 4*B*).

In marked contrast, in the AIC, only rats that used the interoceptive discriminative effects of PTZ to guide their behavior demonstrated higher zif268 mRNA levels than the other groups, which did not differ from each other (main effect of group: *F*_(5,31)_ = 5.68, *p* < 0.001, *pη*^2^ = 0.48, post hoc comparisons: PTZ-trained-Active-PTZ vs each of the other groups vs every other group: *p*s < 0.04; Fig. 4*C*).

No such differences were observed in the BLA (main effect of group: *F*_(5,31)_ = 0.85, *p* = 0.52, *pη*^2^ = 0.12; Fig. 4*D*) or the CeA in which only for the LightCue group differed from the Passive-VEH and PTZ-trained-Active-VEH groups (main effect of group: *F*_(5,31)_ = 3.19, *p* = 0.019, *pη*^2^ = 0.36, post hoc comparisons: *p*s < 0.03; Fig. 4*E*).

### Persistence in discrimination predicts zif268 mRNA levels across the insula

**Figure 5.**
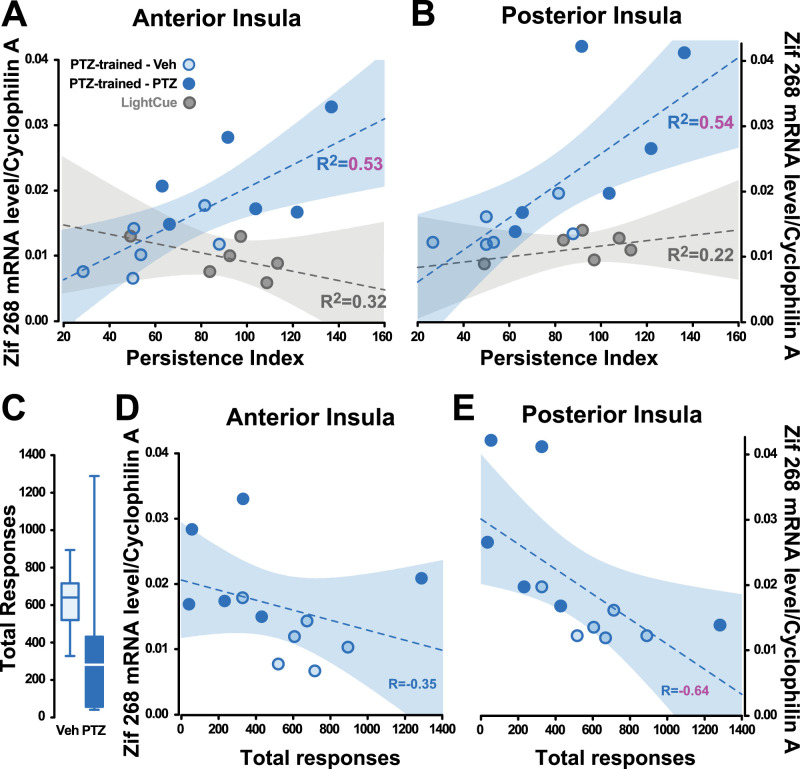
Persistence in discrimination predicts zif268 mRNA levels across the insula. ***A***, ***B***, Zif268 mRNA levels in both the AIC and PIC of the PTZ-trained rats were predicted by the degree of persistence of responding on the correct lever (*R*^2^ = 0.53, *p* < 0.01 and *R*^2^ = 0.54, *p* < 0.01, respectively) as opposed to those trained to rely on an exteroceptive discriminative stimulus to guide their behavior (LightCue-trained rats) (*R*^2^ = 0.32, *p* = 0.24 and *R*^2^ = 0.22, *p* = 0.34 in the AIC and PIC, respectively). ***C***, The challenge condition in PTZ-trained rats, namely, a VEH or a PTZ administration, had no influence on total instrumental output (AL + IL presses) during the extinction session ([main effect of drug: *F*_(1,10)_ = 1.22, *p* = 0.294, p*η*^2^ = 0.11). ***D****–**E***, Total instrumental responding was not positively correlated with Zif268 mRNA levels in the AIC (***D***) or the PIC (***E***; Pearson's correlations: *R* = −0.35, *p* = 0.265 and *R* = −0.64, *p* < 0.05, respectively).

Not only did the utilization of PTZ interoceptive discriminative stimulus properties result in a selective increase in zif268 mRNA levels in the AIC, but the persistence of responding on the correct lever at test was predictive of the magnitude of that increase (*R*^2^ = 0.53, *p* < 0.01; Fig. 5*A*). Zif268 mRNA levels in the PIC, which were highly correlated to those in the AIC (*R* = 0.85, *p* < 0.001, data not shown), were also predicted by the persistence of responding on the correct lever at test in rats trained to use interoceptive discriminative stimuli (*R*^2^ = 0.54, *p* < 0.01; Fig. 5*B*). These dimensional relationships were not observed in equally performing LightCue-trained (*R*^2^ = 0.32, *p* = 0.24 and *R*^2^ = 0.22, *p* = 0.34 in the AIC and PIC, respectively; Fig. 5*A*,*B*) or UnCued rats (*R*^2^ = 0.008, *p* = 0.84 and *R*^2^ = 0.12, *p* = 0.43 in the AIC and PIC, respectively, data not shown). These dimensional relationships cannot be attributable either to a difference in behavioral output as the total lever presses (AL + IL) emitted during the entire challenge session did not differ between rats that used the discriminative stimulus properties of PTZ and those that used that of Vehicle (main effect of drug: *F*_(1,10)_ = 1.22, *p* = 0.294, *pη*^2^ = 0.11; Fig. 5*C*), nor was it positively correlated with zif268 mRNA levels in the AIC (*R* = −0.35, *p* = 0.265; Fig. 5*D*) or the PIC of rats that used interoceptive discriminative stimuli to guide their instrumental responding at test (*R* = −0.64, *p* < 0.05; Fig. 5*E*).

## Discussion

The present data show that rats trained to use the peripherally or centrally generated interoceptive states produced by ISO or PTZ injection, respectively, learnt to guide their instrumental behavior as readily as did their counterparts using exteroceptive light cues. Thereby, this demonstrates that peripherally and centrally generated interoceptive states are as effective “occasion setters” as exteroceptive stimuli. In addition, the analysis of the mRNA levels of the plasticity marker zif268, which were not directly or nonspecifically influenced by the GABA_A_ receptor antagonist PTZ, shows that the use of interoceptive, as opposed to exteroceptive discriminative stimuli to guide behavior, was uniquely associated with functional engagement of the AIC. While the PIC was engaged in response to the passive experience of the effects of PTZ as well as when rats used their interoceptive state as a discriminative stimulus to guide their instrumental behavior, the AIC was only recruited in the latter condition. In marked contrast, zif268 mRNA levels were not elevated in the AIC of rats that performed equally well in a discrimination task guided by exteroceptive cues, thereby showing that the AIC is selectively involved during the use of interoceptive pavlovian discriminative stimuli. This is in agreement with previous evidence in rodents for the role of the IC in guiding goal-directed behaviors and the discriminative stimulus properties of alcohol (Jaramillo et al., 2018; Talpir and Livneh, 2024). Our findings are also consistent with observations in humans that attention to exteroceptive signals does not recruit the AIC (Wang et al., 2019) and that transcranial magnetic stimulation of the AIC alters specifically interoceptive accuracy (Pollatos et al., 2016).

Together, our results demonstrate a hierarchical recruitment of the IC in interoceptive control over behavior in rats. The PIC is engaged during the experience of an internal state, while the AIC is recruited proportionally (as revealed by the correlation between AIC and PIC zif268 mRNA levels in PTZ-trained rats) when the same internal states are used to guide instrumental behavior.

The hierarchical recruitment of the IC shown here in rats to be associated with using internal states to guide motivated behaviors provides functional significance to neural tract-tracing data showing more intra-insular outputs from the PIC than from the AIC (Gehrlach et al., 2020) and electrophysiological recordings supporting a caudal-to-rostral flow of information in the IC (Fujita et al., 2010; Nieuwenhuys, 2012). This observation bridges a gap with human studies showing a functional gradient from the PIC to the AIC in various investigations of interoception and interoceptive control over behavior (Lovero et al., 2009; Kurth et al., 2010; Simmons et al., 2013; Hassanpour et al., 2016). It resonates particularly well with the study by Hassanpour et al. (2016) that demonstrated using functional imaging in humans that the PIC responds directly to the effects of a systemic challenge with ISO while the AIC responds in anticipation of the expected effects. The present results further indicate that the AIC is both functionally recruited in anticipation of future interoceptive states, and when they are used to guide motivated behavior, in line with the insula hierarchical network architecture for active interoceptive inference proposed by Fermin and colleagues (Fermin et al., 2022).

Together, our present data and those of Hassanpour et al. (2016) lend support to the view that interoception is a bidirectional brain–body communication system in which internal states are as much the product of peripherally generated bottom-up physiological signals integrated in the IC as of top-down recruitment of, and control over, these signals (Berntson and Khalsa, 2021; Chen et al., 2021; Hsueh et al., 2023). Furthermore, in the present study, both centrally acting PTZ and peripherally acting ISO supported discrimination learning in the DDT. While ISO exerts strong positive chronotropic effects originating from the periphery, the discriminative properties of PTZ depend on its centrally mediated anxiogenic effects (Vellucci et al., 1988; Carey and Fry, 1993). These findings are also consistent with the somatic marker hypothesis (Damasio, 1996), according to which signals from the body, as well as their externally or internally generated central representations, influence affective behavior, motivation, and executive functions such as decision-making.

As compared with the UnCued group, which had no interoceptive or exteroceptive discriminative stimulus to guide their instrumental behavior and hence performed at chance during the challenge session, rats trained to use a cue light or the discriminative stimulus properties of PTZ versus VEH allocated their instrumental response to the correct lever for a long period of time under extinction conditions. In particular, while PTZ-trained rats challenged under the effects of VEH did not differ in their discrimination performance from those challenged under the effects of PTZ during the first period of the challenge session, they progressively switched their response from the VEH-paired lever to the PTZ-paired lever toward the end of the session. In contrast, the PTZ-trained-PTZ-challenged rats and those that used the exteroceptive Cue light to guide their behavior persisted on the correct lever. These results suggest that a PTZ-evoked internal state is more salient than that evoked by vehicle and that more attention is allocated to the PTZ-generated interoceptive state, thereby promoting a more robust and long-lasting reliance on its discriminative stimulus function to guide behavior.

Together with the evidence that the persistence of responding on the correct lever in the discrimination challenge was predictive of zif268 mRNA levels in the IC, these results are consistent with findings showing that attention to internal signals (such as heart rate) in tasks of interoceptive accuracy correlates with activation of the AIC, which is a core component of the salience network (Chao et al., 2023; Menon et al., 2023) and that the degree of activation of the AIC is correlated with interoceptive accuracy (Critchley et al., 2002, 2004; Wang et al., 2019).

On the other hand, the behavior of mammals facing unexpected reward omission, such as during the extinction challenge sessions in the present study, is accompanied by an aversive emotional state referred to as frustrative nonreward (Amsel, 1992; Papini, 2006). The build-up of such a negative emotional state during the challenge session may result in PTZ-trained-Active-VEH rats progressively generalizing the PTZ-induced state they have learnt to use as a discriminative stimulus to those experienced because of reward omission-induced frustration toward the second half of the extinction challenge, which would result in a switch from a response initially allocated to the VEH-paired lever to switch to the PTZ-paired lever, as observed here.

It was surprising not to observe the same increase in zif268 mRNA levels in the AIC of PTZ-trained rats challenged with VEH as that shown by those challenged with PTZ since they both initially performed equally well. One possible explanation is that in PTZ-trained rats challenged under PTZ, the AIC may undergo stereotyped activity patterns reflecting task-dependent goal-directed reward anticipation during extinction (Livneh et al., 2020; Talpir and Livneh, 2024), whereas PTZ-trained rats challenged under VEH do not recruit such an activity pattern because the VEH-induced state is insufficiently salient. It may, therefore, be considered that drug discrimination performance under the present conditions relies on the contrast between a salient internal state produced by PTZ and a “nonsalient” state produced by VEH, and not the discrimination between two defined salient states. Further research is necessary to elucidate the nature of the computations taking place in the AIC that support interoceptive control of motivated instrumental behavior. 

While the present results identify the IC as a key neural locus associated with the behavioral effects of interoceptive discriminative stimuli, the utilization of the discriminative stimulus properties of PTZ had previously been shown causally to involve the amygdala (Vellucci et al., 1988). Interestingly, CeA or BLA zif268 mRNA levels did not differ between UnCued rats and those that used either exteroceptive or interoceptive discriminative stimuli to guide their instrumental behavior. This may reflect that the amygdala, which is involved in the acquisition and behavioral expression of pavlovian associations (Cador et al., 1989; Parkinson et al., 2000; Everitt et al., 2003), motivational control of instrumental behavior (Balleine et al., 2003; Belin et al., 2009; Malvaez et al., 2015), including by discriminative stimuli (Vellucci et al., 1988; Yun and Fields, 2003) may be active, as would be reflected in increased c-*fos* mRNA levels, but not undergoing zif268-mediated cellular plasticity, when discriminative stimuli are used to guide instrumental behavior after extended training, as is the case in the present experiments. Further research is necessary to delineate the circuit mechanisms by which the amygdala-insular systems contribute to the influence of interoceptive discriminative stimuli on instrumental behavior and whether they are similar between males and females. Indeed, interoceptive abilities and insular responses to interoceptive challenges differ between sexes/genders (Krause-Sorio et al., 2022; Prentice and Murphy, 2022), even though no sex/gender differences were established in the hierarchical functional recruitment of the insula during cardiorespiratory interoceptive stimulation in humans (Hassanpour et al., 2016), to which the present results relate the most. Similarly, since the response of the insula to isoproterenol-induced cardiorespiratory interoceptive stimulation in humans or pharmacologically induced baroreceptor stimulation in anesthetized rats is lateralized (Zhang and Oppenheimer, 1997; Hassanpour et al., 2016), further research is also necessary to determine if it is also the case in the context of the utilization of interoceptive discriminative stimuli to guide instrumental behavior.

In conclusion, the present study demonstrates that both peripherally and centrally generated interoceptive states can be used as discriminative stimuli by rats as effectively as exteroceptive cues to guide their instrumental behavior, albeit via different underlying neural systems. Central detection of internal states and the utilization of these internal states as discriminative stimuli to guide instrumental behavior engage cellular plasticity in the PIC and the AIC, respectively, thereby demonstrating a hierarchical functional organization of the IC in rats as has been shown in humans. Taken together, our data provide clear evidence for the conservation of the hierarchical functional organization of the IC across species.

## Synthesis

Reviewing Editor: Mihaela Iordanova, Concordia University - Loyola Campus Decisions are customarily a result of the Reviewing Editor and the peer reviewers coming together and discussing their recommendations until a consensus is reached. When revisions are invited, a fact-based synthesis statement explaining their decision and outlining what is needed to prepare a revision will be listed below. The following reviewer(s) agreed to reveal their identity: NONE. Note: If this manuscript was transferred from JNeurosci and a decision was made to accept the manuscript without peer review, a brief statement to this effect will instead be what is listed below.

The paper was assessed by the reviewers and reviewing editor and there was clear interest in the studies reported. During the discussion the reviewers raised a number of concerns that are detailed below and need to be addressed in a revision.

1. Further clarity is needed on the DDT to better understand the behavioural and neural data. Clarifying how the drugs were delivered to ensure discrimination remains somewhat opaque, yet it is the backbone of the paper. As other readers may find this difficult to decipher, the authors should clarify this methodological aspect better in the manuscript.

2. The figures would benefit from a clearer representation of the data depicted. For example in Fig 2 the y-axis states 'learning to criterion' but it's unclear how that is related to the numbers of that axis. Fig 3B refers to two 6-minute time bins but there is insufficient information on the figure to understand where those bins come from and what they mean. The graphical representation of injections, and cues above the panels along with the misaligned word description below the panel is confusing.

3. The manuscript makes excessive use of acronyms which hinder comprehension and readability. For example %ALP is not a meaningful acronym and requires the reader to dig into the text to understand the figure. As there is on word limit, removing unnecessary acronyms would improve the paper. Similarly, 'PTZ-trained-Active_VEH' is not a very intuitive name for a group. Please revise these.

4. The exclusive focus on male rats is a limitation given known sex differences in the perception and processing of internal states (see Prentice F, Murphy J (2022): Sex differences in interoceptive accuracy: A meta-analysis. Neurosci Biobehav Rev 132: 497-518). This should be acknowledged in the title and discussed in the manuscript as this speaks to the generalizability the findings across sexes.

5. Given evidence for lateralized function of the insula exists, did the authors examine whether there were differences between the left and right hemispheres? Assessing this would help better understand the involvement of the insula in the present task.

6. The authors note that isoproterenol treatment had to be discontinued due to side effects. The manuscript should include a detailed description of these specific side effects. Additionally, the authors should provide a justification that these adverse effects did not confound the behavioral outcomes reported. It is also important to clarify that the side effects did not raise concerns about distinguishing peripheral from central interoceptive contributions in the instrumental behavior observed.

7. The authors did not report any change in activity in the BLA and explain that due to Pavlovian vs instrumental conditioning. However, the Wassum lab has implicated the BLA in instrumental conditioning so a more nuanced account of these data is needed.

## Author Response

We would like to thank the reviewers and the reviewing editor for their overall positive feedback on our manuscript and their constructive comments We have also merged the results originally displayed in SOM Figure 1 with Figure 4 in order to avoid a SOM, as requested by the journal's editorial team We believe the manuscript has been greatly enhanced in this process, and we thank, once again, the reviewers for their astute and constructive comments.

Detailed response to the reviewers:

1. Further clarity is needed on the DDT to better understand the behavioural and neural data. Clarifying how the drugs were delivered to ensure discrimination remains somewhat opaque, yet it is the backbone of the paper. As other readers may find this difficult to decipher, the authors should clarify this methodological aspect better in the manuscript. ⇒ we carefully verified the methods of the original manuscript and this section describes, we believe, very clearly how the drugs were delivered. We have added the route of administration to this section to ensure the reader has all the relevant information in the same section: "The rats then progressed onto drug discrimination training, which consisted of forty-four, 12-minute daily sessions, 6-7 days per week. In these sessions, both levers were presented, but responding on only one of these levers, the active lever, which was pseudo-randomly assigned from one session to another, was reinforced. For a rat to respond on the correct lever on any given session, it had to use the discriminative properties of an interoceptive stimulus (PTZ, n=12, or ISO, n=12, vs VEH) administered intraperitoneally 20 minutes before the start of the session or that of an exteroceptive white light cue (LightCue) illuminated above the active lever throughout the session. A specific interoceptive discriminative stimulus was always paired with the same active lever, randomly allocated across individuals (Figure 1). For each independent group, the different discrimination conditions (PTZ vs VEH, ISO vs VEH or LightCue vs no cue) were alternated every other day for the first 10 sessions, after which a pseudo-random sequence was employed so that PTZ or ISO were never injected on two consecutive days, but never more than four sessions apart".

2. The figures would benefit from a clearer representation of the data depicted. For example in Fig 2 the y-axis states 'learning to criterion' but it's unclear how that is related to the numbers of that axis. Fig 3B refers to two 6-minute time bins but there is insufficient information on the figure to understand where those bins come from and what they mean. The graphical representation of injections, and cues above the panels along with the misaligned word description below the panel is confusing. ⇒ We have amended the figures accordingly. In Figure 2, we have relabelled the title of the Y axis to 'discrimination index' and provided a clear explanation of what it represents in the legend. In Figure 3, we removed the representation of injections and cues and amended the overall layout to improve clarity. Across legends, we removed all acronyms and referred only to the treatment groups.

3. The manuscript makes excessive use of acronyms which hinder comprehension and readability. For example %ALP is not a meaningful acronym and requires the reader to dig into the text to understand the figure. As there is on word limit, removing unnecessary acronyms would improve the paper. Similarly, 'PTZ-trained-Active_VEH' is not a very intuitive name for a group. Please revise these. ⇒ Except for the drugs and treatment groups, the names of which we have simplified, we have removed all acronyms. Additionally, we have included a blurb explaining the "Active"/ "Passive" terminology used in the figures under the Procedures section of the Materials and Methods: "Forty-five minutes after this challenge session, rats were sacrificed, and their brains were harvested fresh, snap-frozen and stored at -80{degree sign}C until processed for qPCR alongside those of an untrained control group. This group (referred to as "Passive", which comprised 12 drug-naïve rats sacrificed forty-five minutes after an intraperitoneal injection of either PTZ (n = 6) or vehicle (n = 6). The PTZ-administered rats of this control group had passively experienced the interoceptive effects of PTZ, unlike the PTZ-trained group (referred to as "Active"), which utilised them as a discriminative stimulus to guide their instrumental reward-related behaviour.

4. The exclusive focus on male rats is a limitation given known sex differences in the perception and processing of internal states (see Prentice F, Murphy J (2022): Sex differences in interoceptive accuracy: A meta-analysis. Neurosci Biobehav Rev 132: 497-518). This should be acknowledged in the title and discussed in the manuscript as this speaks to the generalizability the findings across sexes. ⇒ Point taken. This is now acknowledged in the title, and a dedicated section has been added to the discussion that reads: ". Further research is necessary to delineate the circuit mechanisms by which the amygdala-insular systems contribute to the influence of interoceptive discriminative stimuli on instrumental behaviour and whether they are similar between males and females. Indeed, interoceptive abilities and insular responses to interoceptive challenges differ between sexes/genders (Krause-Sorio et al., 2022; Prentice and Murphy, 2022), even though no sex/gender differences were established in the hierarchical functional recruitment of the insula during cardiorespiratory interoceptive stimulation in humans (Hassanpour et al., 2016), to which the present results relate the most.

5. Given evidence for lateralized function of the insula exists, did the authors examine whether there were differences between the left and right hemispheres? Assessing this would help better understand the involvement of the insula in the present task. ⇒ This is another excellent suggestion for follow-up studies. As described in the methods, we pooled the µ-punches of the brain regions we investigated, which prevents us from investigating any lateralisation effect. We have nevertheless addressed this point in the discussion of our revised manuscript, which reads as follows: "Similarly, since the response of the insula to isoproterenol-induced cardiorespiratory interoceptive stimulation in humans or pharmacologically-induced baroreceptor stimulation in anaesthetised rats is lateralised (Zhang and Oppenheimer, 1997; Hassanpour et al., 2016), further research is also necessary to determine if it is also the case in the context of the utilisation of interoceptive discriminative stimuli to guide instrumental behaviour." 6. The authors note that isoproterenol treatment had to be discontinued due to side effects. The manuscript should include a detailed description of these specific side effects. Additionally, the authors should provide a justification that these adverse effects did not confound the behavioral outcomes reported. It is also important to clarify that the side effects did not raise concerns about distinguishing peripheral from central interoceptive contributions in the instrumental behavior observed. ⇒ We reported that the use of this drug had to be discontinued "because of a suspected adverse side effect in one rat", not an established side effect.

Actually, one rat was found dead in this experiment, and the nature of animal experimentation legislation in the UK is such that, despite no evidence and a non-conclusive post-mortem investigation, the death was attributed to that animal having received isoproterenol on the ground that isoproterenol can have negative cardiovascular effects in rats. We were forced to interrupt the administration of the drug, and to ensure that the behavioural outcomes reported here were not confounded by any non-interoceptive effect of isoproterenol, rats whose body weight had not increased in the previous week were excluded. It remains the case that this case is an excellent opportunity to demonstrate the loss of discrimination in a group that had clearly acquired it with a drug that does not cross the blood-brain barrier fast enough to have any central effects at the time animals were instrumentally trained.

We edited the relevant section of the manuscript accordingly.

7. The authors did not report any change in activity in the BLA and explain that due to Pavlovian vs instrumental conditioning. However, the Wassum lab has implicated the BLA in instrumental conditioning so a more nuanced account of these data is needed. ⇒ Point well taken. This discussion was not simply about contrasting the role of the amygdala in Pavlovian vs instrumental conditioning, as it referred to the fact that when the behaviour is overtrained, it may no longer be dependent on plasticity in the amygdala. Nevertheless, we have rewritten the relevant section of the discussion. It now reads as follows: "This may reflect that the amygdala, which is involved in the acquisition and behavioural expression of Pavlovian associations (Cador et al., 1989; Parkinson et al., 2000; Everitt et al., 2003), motivational control of instrumental behaviour (Balleine et al., 2003; Belin et al., 2009; Malvaez et al., 2015), including by discriminative stimuli (Vellucci et al., 1988; Yun and Fields, 2003) may be active, as would be reflected in increased c-fos mRNA levels, but not undergoing zif268-mediated cellular plasticity, when discriminative stimuli are used to guide instrumental behaviour after extended training, as is the case in the present experiments.
